# Nosocomial *Escherichia coli*, *Klebsiella pneumoniae*, *Pseudomonas aeruginosa*, and *Staphylococcus aureus*: Sensitivity to Chlorhexidine-Based Biocides and Prevalence of Efflux Pump Genes

**DOI:** 10.3390/ijms26010355

**Published:** 2025-01-03

**Authors:** Marina V. Kuznetsova, Larisa Y. Nesterova, Veronika S. Mihailovskaya, Polina A. Selivanova, Darja A. Kochergina, Marina O. Karipova, Igor V. Valtsifer, Anastasia S. Averkina, Marjanca Starčič Erjavec

**Affiliations:** 1 Laboratory of Molecular Biotechnology, Institute of Ecology and Genetics of Microorganisms Ural Branch Russian Academy of Sciences, Perm Federal Research Centre of Ural Branch of RAS, 614081 Perm, Russia; mar@iegm.ru (M.V.K.); veranikamihailovskaja@yandex.ru (V.S.M.); polina.selivanova2003@gmail.com (P.A.S.); kocdas@ya.ru (D.A.K.); 2 Department of Microbiology and Virology, Perm State Medical University Named After Academician E. A. Wagner, 614000 Perm, Russia; mari.karipova@yandex.ru; 3 Laboratory of Microorganisms’ Adaptation, Institute of Ecology and Genetics of Microorganisms Ural Branch Russian Academy of Sciences, Perm Federal Research Centre of Ural Branch of RAS, 614081 Perm, Russia; larisa.nesterova@bk.ru; 4 Department of Multiphase Dispersed System, Institute of Technical Chemistry Ural Branch Russian Academy of Sciences, Perm Federal Research Centre of Ural Branch of RAS, 614013 Perm, Russia; igor12381@mail.ru (I.V.V.); anastasiya.av11@yandex.ru (A.S.A.); 5 Department of Microbiology, Biotechnical Faculty, University of Ljubljana, 1000 Ljubljana, Slovenia; 6 Department of Biology, Faculty of Natural Sciences and Mathematics, University of Maribor, 2000 Maribor, Slovenia

**Keywords:** *Escherichia coli*, *Klebsiella pneumoniae*, *Pseudomonas aeruginosa*, *Staphylococcus aureus*, biocides, chlorhexidine, quaternary ammonium compounds, antimicrobial resistance, biofilm formation, efflux pumps

## Abstract

The widespread use of disinfectants and antiseptics has led to the emergence of nosocomial pathogens that are less sensitive to these agents, which in combination with multidrug resistance (MDR) can pose a significant epidemiologic risk. We investigated the susceptibility of nosocomial *Escherichia coli*, *Klebsiella pneumoniae*, *Pseudomonas aeruginosa*, and *Staphylococcus aureus* to a 0.05% chlorhexidine (CHX) solution and a biocidal S7 composite solution based on CHX (0.07%) and benzalkonium chloride (BAC, 0.055%). The prevalence of efflux pump genes associated with biocide resistance and their relationship to antibiotic resistance was also determined. Both biocides were more effective against Gram-positive *S. aureus* than Gram-negative bacteria. The most resistant strains were *P. aeruginosa* strains, which were mainly killed by 0.0016% CHX and by 0.0000084% (CHX)/0.0000066% (BAC) S7. The S7 bactericidal effect was observed on *P. aeruginosa* and *S. aureus* after 10 min, while the bactericidal effect of CHX was only observed after 30 min. *qacEΔ1* and *qacE* efflux pump genes were prevalent among *E. coli* and *K. pneumoniae*, while *mexB* was more often detected in *P. aeruginosa*. *norA*, *norB*, *mepA*, *mdeA*, and *sepA* were prevalent in *S. aureus*. The observed prevalence of efflux pump genes highlights the potential problem whereby the sensitivity of bacteria to biocides could decline rapidly in the future.

## 1. Introduction

In recent years, the problem of increasing antimicrobial resistance among opportunistic and pathogenic microorganisms has become acute, as the prevalence of multidrug resistant (MDR) strains has increased [1,2,3]. An official World Health Organization multicenter study on the prevalence of nosocomial infections conducted in 50 clinics in 14 countries, representing four WHO regions (Europe, Eastern Mediterranean, South-East Asia and Western Pacific) found that 8.7% of hospitalized patients, i.e., over 1.4 million people worldwide, had nosocomial infections [4]. The treatment of such infections requires additional diagnostic and therapeutic measures, prolonging the duration of the patient’s hospital stay and leading to significant economic costs. In addition, nosocomial infections lead to a significant deterioration in the quality of life and increase the risk of an unfavorable course of the disease [5,6]. According to the WHO recommendations from 2024, the list of microorganisms that pose the greatest threat to society has expanded considerably. However, *Escherichia coli*, *Klebsiella pneumoniae*, *Pseudomonas aeruginosa*, and *Staphylococcus aureus* remain with high priority, since infections caused by multi- and pan-resistant forms of these bacteria are associated with high patient mortality [7].

The problem of nosocomial infections of patients due to contaminated environmental surfaces is of utmost importance for hospitals, especially for intensive care units [5,8]. Therefore, the treatment of biotic (hands of medical staff) and abiotic (medical equipment) surfaces with various antiseptics and disinfectants is a necessary part of the infection control program and prevention of nosocomial infections [9,10]. The WHO Model List of Essential Medicines (EML) 2021 [11] includes essential products as disinfectants, most of which contain chlorhexidine (CHX), a biguanide derivative. In addition, many other products, such as alcohols, iodine, povidone iodine, and quaternary ammonium compounds (QACs) are used. CHX is a lipophilic and positively charged compound and can therefore interact with negatively charged phospholipids and lipopolysaccharides of the bacterial cell wall and outer membrane. Low concentrations of this compound impair membrane integrity, whereas high concentrations cause the congealing of cytoplasm, which ultimately leads to cell death [12]. QACs, which are often used as disinfectants, can attach to the bacterial cell wall due to their positive charge, which causes cell lysis. However, the widespread use of antiseptics and disinfectants (including in public facilities and healthcare settings during the COVID-19 pandemic) has led to the emergence of resistant strains of microorganisms, which, combined with multidrug resistance, can pose a significant threat [13].

One of the main mechanisms of bacterial resistance to biocides is the expression of efflux pumps. There are six families of efflux systems: major facilitator superfamily (MFS), multidrug and toxic compound extrusion (MATE) superfamily, ATP-binding cassette (ABC) superfamily, small multidrug resistance (SMR) superfamily, resistance nodulation and cell division (RND) superfamily, and the proteobacterial antimicrobial compound efflux (PACE) superfamily [14]. Most of them are able to transport various antibacterial substances out of the cell. For example, the AcrAB-TolC multidrug RND efflux pump system controls the efflux of antibiotics and biocides, including CHX [15]. The QacE and QacEΔ1 efflux pumps (SMR superfamily) can efflux QACs and CHX, and their overexpression has been associated with increased CHX MIC in *E. coli* biofilms [16]. The MFS family includes EmrAB-TolC and QacA/B, the latter of which can mediate the transport of antibiotics and biocides such as CHX and QACs in staphylococci [17]. Importantly, efflux pump genes associated with biocide resistance can be located not only on the chromosome (*emrE*, *mdfA*, *sugE*, *ydgE*, and *ydgF*), but also on mobile genetic elements such as plasmids, integrons, and transposons (*oqxA*, *oqxB*, *qacEΔ1*, *qacE*, *qacF/H/I*, *qacG*, and *sugE*) [18]. Moreover, efflux pump genes can be localized on the same mobile elements as antibiotic resistance genes, leading to cross- or co-resistance to antiseptics, disinfectants, and antimicrobials [18]. In addition, bacteria form biofilms on surfaces, making them more resistant to biocides [19].

The aim of this study was to evaluate the susceptibility of nosocomial strains of *E. coli*, *K. pneumoniae*, *P. aeruginosa*, and *S. aureus* to CHX and to S7, a biocidal composition based on CHX and the cationic surfactant benzalkonium chloride (BAC), and to assess the prevalence of efflux pump genes associated with biocide resistance and to determine their association with the MDR bacterial phenotype.

## 2. Results

### 2.1. Assessment of Sensitivity to Biocidal Compositions and Detection of Efflux Pump Genes in Nosocomial E. coli

#### 2.1.1. Sensitivity of *E. coli* Strains to Chlorhexidine-Based Biocidal Compositions

**Figure 1:**
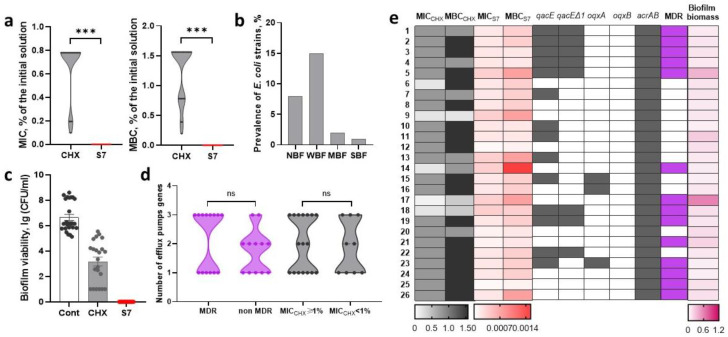
Sensitivity of *E. coli* strains to biocidal compositions based on chlorhexidine. (**a**) Minimum inhibitory (MIC) and minimum bactericidal (MBC) concentrations of chlorhexidine (CHX) and the S7 composite solution of CHX (0.07%) and the cationic surfactant benzalkonium chloride (BAC, 0.055%) (S7). MIC and MBC are presented as a percentage of dilution of the initial solutions. (**b**) The prevalence of non-biofilm-forming (NBF), weak biofilm-forming (WBF), moderate biofilm-forming (MBF) and strong biofilm-forming (SBF) strains. (**c**) The viability of *E. coli* after the addition of CHX or S7 to the one-day old biofilm. (**d**) The prevalence of *E. coli* containing a certain number of efflux pump genes among MDR and non-MDR strains with MBC_CHX_ ≥ 1% and MBC_CHX_ < 1%. (**e**) Heat map depicting the obtained MIC and MBC results and presence of the tested efflux pump genes in relation to MDR and biofilm biomass. 1–26, *E. coli* strains EC1-EC26. Data are mean ± standard error of the mean (SEM), n indicates independent strains, *** *p* < 0.0005.

For the analyzed *E. coli* strains, in the case of the CHX standard solution, the median MIC_CHX_ and MBC_CHX_ were 0.78% of the initial 0.05% CHX solution (which corresponded to a final concentration of CHX 0.00039%, i.e., 3.9 µg/mL) and 1.56% (0.00078%, i.e., 7.8 µg/mL), respectively. In the case of the S7 composite solution, the MIC_S7_ and MBC_S7_ values were 0.00019% (or 0.00000013/0.00000010%, i.e., 0.0013/0.0010 µg/mL CHX/BAC) and 0.00038% (or 0.00000027/0.00000021%, i.e., 0.0027/0.0021 µg/mL CHX/BAC) of the initial solution, respectively (Figure 1a). Consequently, *E. coli* strains were more sensitive to the S7 composite solution than to the CHX solution.

When evaluating the effect of the CHX standard solution and the S7 composite solution on the one-day old biofilm (the median biomass of the daily biofilm was 0.123 OD_570_ units), 80.8% (21/26) of the *E. coli* strains (in the range from 3.6 × 10^2^ to 2.8 × 10^5^ CFU/mL) in the biofilm survived after CHX, while S7 had a bactericidal effect on all strains in the biofilm (Figure 1c). There was no correlation between biofilm biomass and survival after exposure to CHX (r = 0.04). No significant correlations were found between the MDR phenotype and the sensitivity to chlorhexidine-based biocidal compositions.

#### 2.1.2. Efflux Pump Genes in *E. coli* Strains

The prevalence of the efflux pump genes *qacE*, *qacEΔ1*, *oqxA*, *oqxB*, and *acrAB* was 53.8 (14/26), 30.8 (8/26), 11.5 (3/26), 0, and 100%, respectively. In general, nosocomial *E. coli* more often carried either one (11/26, 42.3%) or three (10/26, 38.4%) of the listed genes, less often two (5/26, 19.2%). To assess the associations of efflux pump gene presence with the sensitivity to tested biocides, the *E. coli* strains were divided into two groups: strains with MBC_CHX_ ≥ 1% of the initial solution and strains with MBC_CHX_ < 1%. With the exception of *oqxA*, which was only found in the group of strains with MBC_CHX_ ≥ 1%, the presence of efflux pump genes did not differ significantly between the two groups of strains (Figure 1d,e). MDR and non-MDR strains did not differ significantly in the total number of efflux pump genes (*p* = 0.27, *t*-test); however, MDR strains more frequently contained three efflux pump genes (Figure 1d). Among the MDR *E. coli*, *qacEΔ1* was significantly more often detected (*p* < 0.05, Fisher’s exact test).

### 2.2. Assessment of Sensitivity to Biocidal Compositions and Detection of Efflux Pump Genes in Nosocomial K. pneumoniae

#### 2.2.1. Sensitivity of *K. pneumoniae* Strains to Chlorhexidine-Based Biocidal Compositions

**Figure 2:**
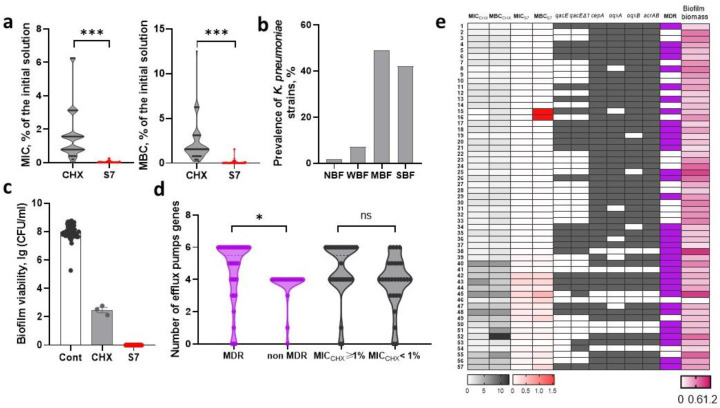
Sensitivity of *K. pneumoniae* strains to biocidal compositions based on chlorhexidine. (**a**) Minimum inhibitory (MIC) and minimum bactericidal (MBC) concentrations of chlorhexidine (CHX) and the S7 composite solution of CHX (0.07%) and the cationic surfactant benzalkonium chloride (BAC, 0.055%) (S7). MIC and MBC are presented as a percentage of dilution of the initial solutions. (**b**) The prevalence of non-biofilm-forming (NBF), weak biofilm-forming (WBF), moderate biofilm-forming (MBF) and strong biofilm-forming (SBF) strains. (**c**) The viability of *E. coli* after addition of CHX or S7 on the one-day old biofilm. (**d**) The prevalence of *E. coli* containing a certain number of efflux pump genes among MDR and non-MDR strains with MBC_CHX_ ≥ 1% and MBC_CHX_ < 1%. (**e**) Heat map depicting the obtained MIC and MBC results and presence of tested efflux pump genes in relation to MDR and biofilm biomass. 1–57, *K. pneumoniae* strains KP1-KP57. Data are mean ± SEM, n indicates independent strains, * *p* < 0.05, *** *p* < 0.0005.

*K. pneumoniae* was characterized by high heterogeneity in sensitivity to biocidal compositions. The ranges of MIC_CHX_ and MBC_CHX_ of the *K. pneumoniae* studied were 0.012–6.25% and 0.195–12.5% of the initial 0.05% CHX solution, respectively (which corresponded to the range of final concentrations of CHX 0.000006–0.00313%, i.e., 0.06–31.3 µg/mL and 0.000098–0.0625%, i.e., 0.98–625 µg/mL). MIC_S7_ varied in the range of 0.003–0.25% (the median was 0.0000084/0.0000066% CHX/BAC, i.e., 0.084/0.066 µg/mL), MBC_S7_ varied in the range of 0.006–1.56% of the initial solution (the median MBC_S7_ was equal to MIC_S7_) (Figure 2a).

**Figure 3:**
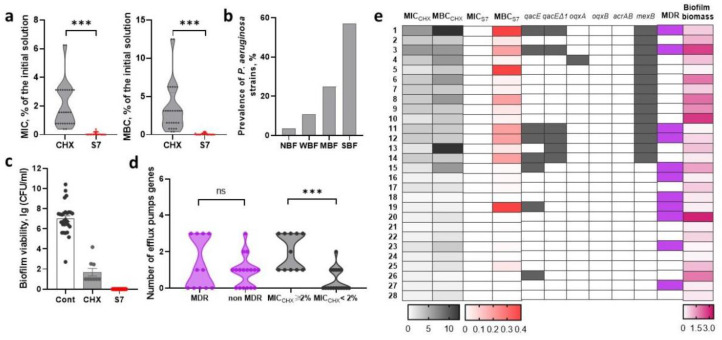
Sensitivity of *P. aeruginosa* strains to biocidal compositions based on chlorhexidine. (**a**) Minimum inhibitory (MIC) and minimum bactericidal (MBC) concentrations of chlorhexidine (CHX) and the S7 composite solution of CHX (0.07%) and the cationic surfactant benzalkonium chloride (BAC, 0.055%) (S7). MIC and MBC are presented as a percentage of dilution of the initial solutions. (**b**) The prevalence of non-biofilm-forming (NBF), weak biofilm-forming (WBF), moderate biofilm-forming (MBF) and strong biofilm-forming (SBF) strains. (**c**) The viability of *E. coli* after addition of CHX or S7 on the one-day old biofilm. (**d**) The prevalence of *E. coli* containing a certain number of efflux pump genes among MDR and non-MDR strains with MBC_CHX_ ≥ 1% and MBC_CHX_ < 1%. (**e**) Heat map depicting the obtained MIC and MBC results and presence of tested efflux pump genes in relation to MDR and biofilm biomass. 1–28, *P. aeruginosa* strains PA1-PA28. Data are mean ± SEM n indicates independent strains, *** *p* < 0.0005.

*K. pneumoniae* strains differed in their ability to form biofilms. The range of OD_570_ values was 0.01–0.995. Most strains, 49.1%, were assigned to the group of moderate biofilm-forming (MBF) strains, while 42.1% of strains were assigned to the group of strong biofilm-forming (SBF) strains. One strain did not form a biofilm, and the remaining 7% of strains were classified as weak biofilm-forming (WBF) strains (Figure 3b). Only 5.3% (3/57) of *K. pneumoniae* strains were able to survive in biofilms after exposure to CHX, while S7 had a bactericidal effect on all strains in biofilms (Figure 2c). In our study, the formation of more massive biofilms did not correlate with the survival of bacteria in biofilms exposed to CHX. It was found that strains resistant to cefotaxime and strains with the MDR phenotype had higher values of MIC_CHX_ and MBC_CHX_ (*p* < 0.05, *t*-test).

#### 2.2.2. Efflux Pump Genes in *K. pneumoniae* Strains

The prevalence of nosocomial *K. pneumoniae* containing various efflux pumps was high. Specifically, 87.7% of strains carried *cepA* and *acrAB* genes, 82.5% carried *oqxB*, 77.2% carried *oqxA*, 47.4% carried *qacEΔ1*, and 42.1% carried *qacE*. Strains with four efflux pump genes (38.6%), as well as strains with all studied efflux pump genes (31.6%), were detected more often. The strains were divided into two groups: strains with MBC_CHX_ ≥ 1% of the initial solution and strains with MBC_CHX_ < 1%. There was no correlation between the number of efflux pump genes or the presence of a particular efflux pump gene and the ability to survive the addition of CHX in concentrations exceeding 1% of the initial solution. However, it is important to note that an association with multidrug resistance was found. MDR strains contained more efflux pump genes per strain than non-MDR *K. pneumoniae* (*p* = 0.042, *t*-test) (Figure 1d). The *qacE* gene was only found among MDR *K. pneumoniae*, and *qacEΔ1* was also significantly more often detected in this group (*p* < 0.05, Fisher’s exact test). The prevalence of other efflux pump genes did not differ between these groups.

### 2.3. Assessment of Sensitivity to Biocidal Compositions and Detection of Efflux Pump Genes in Nosocomial P. aeruginosa

#### 2.3.1. Sensitivity of *P. aeruginosa* Strains to Chlorhexidine-Based Biocidal Compositions

For *P. aeruginosa* strains, the MIC_CHX_ values were in the range of 0.39–6.25% for the initial 0.05% CHX solution (which corresponded to the range of final concentrations of CHX 0.000195–0.003125, i.e., 1.95–31.3 µg/mL). To achieve a bactericidal effect, the CHX concentration had to be increased by an average of 2-fold: MBC_CHX_ ranged from 3.125 to 12.5% for the initial CHX solution (0.000195–0.00625%, i.e., 1.95–62.5 µg/mL). Similar, to previous results, the *P. aeruginosa* strains showed greater sensitivity to the S7 composite solution than to CHX: the medians MIC_S7_ and MBC_S7_ corresponded to 0.024% of the initial solution (or 0.000017/0.000013% CHX/BAC, i.e., 0.17/0.13 µg/mL) (*p* < 0.001, *t*-test) (Figure 3a).

*P. aeruginosa* strains were characterized by high variability in their ability to form biofilms (OD_570_ values ranged from 0.106 to 3.125), while most strains were classified as SBF (Figure 3b). Here, 32% (9/28) of *P. aeruginosa* remained viable when exposed to CHX on the formed biofilms, and the average survival rate was (5.6 ± 10) × 10^2^ CFU/mL. S7 had a bactericidal effect on all strains in biofilms (Figure 3c). No significant correlation was found between the ability to form biofilms and survival in biofilms after exposure to biocides (r = 0.15). No significant correlation was found between MDR phenotype and sensitivity to chlorhexidine-based biocidal compositions.

#### 2.3.2. Efflux Pump Genes in *P. aeruginosa* Strains

Among the studied efflux pump genes, membrane multidrug exporter gene *mexB* was the most common, being present in 50% of strains. The efflux pump genes *qacE*, *qacEΔ1*, and *oqxA* were detected in 28.6%, 21.4%, and 3.6% of strains, respectively. *oqxB* and *acrAB* were not identified. *P. aeruginosa* strains were divided into the following groups according to their sensitivity to CHX: strains with MBC_CHX_ < 2% and strains with MBC_CHX_ ≥ 2%. The strains with MBC_CHX_ ≥ 2% contained more efflux pump genes (*p* < 0.001, *t*-test) and carried the *qacE* gene significantly more often (*p* < 0.05, Fisher’s exact test). The *qacEΔ1* gene was only detected in this group. The latter efflux pump gene was only detected in the group of strains with MBC_CHX_ ≥ 2%. Among the MDR *P. aeruginosa* strains, the *qacE* gene was significantly more often detected (*p* < 0.05, Fisher’s exact test). The prevalence of other genes did not differ between MDR and non-MDR strains; however, three efflux pump genes were more often harbored in MDR strains (Figure 3d).

### 2.4. Assessment of Sensitivity to Biocidal Compositions and Detection of Efflux Pump Genes in Nosocomial S. aureus

#### 2.4.1. Sensitivity of *S. aureus* Strains to Chlorhexidine-Based Biocidal Compositions

**Figure 4:**
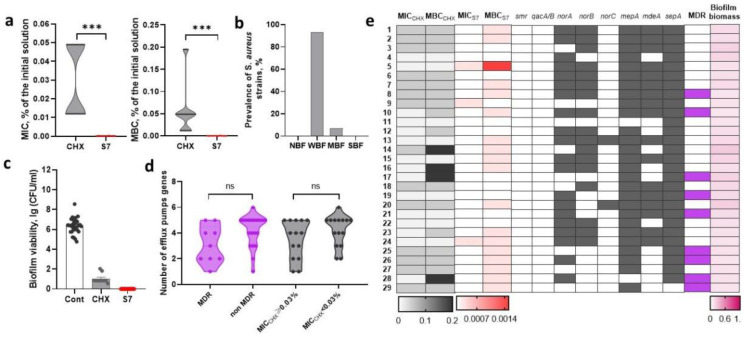
Sensitivity of *S. aureus* strains to biocidal compositions based on chlorhexidine. (**a**) Minimum inhibitory (MIC) and minimum bactericidal (MBC) concentrations of chlorhexidine (CHX) and the S7 composite solution of CHX (0.07%) and the cationic surfactant benzalkonium chloride (BAC, 0.055%) (S7). MIC and MBC are presented as a percentage of dilution of the initial solutions. (**b**) The prevalence of non-biofilm-forming (NBF), weak biofilm-forming (WBF), moderate biofilm-forming (MBF) and strong biofilm-forming (SBF) strains. (**c**) The viability of *E. coli* after addition of CHX or S7 on the one-day old biofilm. (**d**) The prevalence of *E. coli* containing a certain number of efflux pump genes among MDR and non-MDR strains with MBC_CHX_ ≥ 1% and MBC_CHX_ < 1%. (**e**) Heat map depicting the obtained MIC and MBC results and the presence of tested efflux pump genes in relation to MDR and biofilm biomass. 1–29, *S. aureus* strains SA1-SA29. Data are mean ± SEM, n indicates independent strains, *** *p* < 0.0005.

The MIC_CHX_ and MBC_CHX_ *S. aureus* strains were in the range of 0.012–0.049% and 0.049–0.195% of the initial 0.05% CHX solution (the medians in terms of final CHX concentrations were 0.000006 and 0.000025%, i.e., 0.06–0.25 µg/mL), respectively. *S. aureus* showed significantly greater sensitivity to S7 than to CHX. The MIC_S7_ value ranged from 0.000188 to 0.000375% (median was 0.000000132%/0.000000103 CHX/BAC, i.e., 0.0013/0.001 µg/mL) (*p* = 0.002, *t*-test) from the original composition, and the MBC_S7_ value ranged from 0.000188 to 0.0015% (median was 0.000000263/0.000000206% CHX/BAC, i.e., 0.0026/0.0021 µg/mL) (*p* < 0.001, *t*-test) (Figure 4a).

The majority of *S. aureus* strains were characterized by a weak ability to form biofilms (median OD_570_ was 0.158), and only two strains were classified as MBF (Figure 4b). After exposure to CHX, 15 *S. aureus* strains remained viable in the biofilm at (8.4 ± 14) × 10^2^ CFU/mL. The use of S7 had a bactericidal effect on all strains (Figure 4c). The biofilm biomass and the survival rate of *S. aureus* in biofilms after exposure to CHX were weakly correlated (r = 0.278). No significant correlation was found between the MDR phenotype and sensitivity to chlorhexidine-based biocidal compositions.

#### 2.4.2. Efflux Pump Genes in *S. aureus* Strains

Most *S. aureus* strains contained the multidrug efflux pump gene *sepA* (28/29, 96.6%) and MATE family efflux pump gene *mepA* (27/29, 93.1%). The *norA*, *norB*, and *mdeA* efflux pump genes were detected in 68.9 (20/29), 58.6, and 58.6% (17/29) of strains, respectively. The *norC* gene was detected in 6.9% (2/29), whereas the *smr* and *qacA/B* genes were not detected. The strains were divided into two groups: strains with MBC_CHX_ = 0.03% and strains with MBC_CHX_ < 0.03% of the initial solution. No correlation was found between the number of efflux pump genes or the presence of a particular efflux pump gene and the ability to survive the addition of CHX at concentrations exceeding 0.03% of the initial solution. Furthermore, no correlation with the MDR phenotype was found (Figure 4d).

### 2.5. Comparison of the Sensitivity of Nosocomial E. coli, K. pneumoniae, P. aeruginosa, and S. aureus to Chlorhexidine-Based Biocidal Compositions in Plankton and Biofilm

Gram-positive bacteria *S. aureus* had the greatest sensitivity to CHX and S7. CHX had a bactericidal effect at its final concentration of 0.000025%, while the effective concentration of CHX decreased by 100-fold when the S7 composite solution was used (Table 1). The bactericidal effect of CHX against *E. coli* and *K. pneumoniae* was similar, averaging 0.0008% (1.56% of the native solution). The most resistant microorganisms were *P. aeruginosa*. Cells were lysed in most cases in 0.0016% CHX (3.125% of the native solution), while the MBC_CHX_ value for a number of strains was 0.003 (6.25% of the native solution). Evaluation of the effect of the biocidal compositions on the formed biofilms showed that bacteria of all species studied were able to survive in biofilms under the action of CHX: 21 of 26 strains of *E. coli* (80.8%), 3 of 57 *K. pneumoniae* (5.3%), 9 of 28 *P. aeruginosa* (32.1%), and 10 of 25 *S. aureus* (34.5%). After exposure to S7, the bacteria did not survive in the biofilms. In our study, the *oqxA* and *oqxB* genes were also detected in a large percentage of cases in *K. pneumoniae* strains (77.2% and 82.5%, respectively), while they were practically absent in *E. coli* (11.5 and 0%, respectively).

### 2.6. Sensitivity of Nosocomial E. coli, K. pneumoniae, P. aeruginosa, and S. aureus to Chlorhexidine-Based Biocidal Compositions on the Surface

The sensitivity of nosocomial bacterial strains to CHX and S7 was tested using an in situ model on “ceramic tiles” and “plastic” surfaces with two variants: those with immediate exposure to biocides (after application and drying of the bacterial suspension on the surface) and those with exposure of biofilms that have formed on the surfaces (a day after application of the bacterial suspension). The contact times of bacteria with biocides in the first variant was 10 min, 30 min and 1 h. In the second variant, the contact times were 1 h and 2 h. When evaluating the immediate effect of biocides, the bactericidal effect of CHX and S7 on *E. coli*, *K. pneumoniae,* and *S. aureus*, applied to the surface of “ceramic tiles” and “plastic”, was observed after a 10-min exposure to the biocide (Table 2). With regard to *P. aeruginosa*, the bactericidal effect of CHX on both surfaces occurred only after 30 min.

After daily exposure of bacterial suspensions on the surfaces, we recorded a high survival rate for *K. pneumoniae*, while *E. coli*, *S. aureus* and, surprisingly, *P. aeruginosa* died during the day when drying on the surface, and their numbers did not exceed 100 CFU/100 cm^2^. Nevertheless, treatment with biocides led to the complete removal of bacteria from the surface (Table 3).

## 3. Discussion

The widespread occurrence of biocide-tolerant bacteria is one of the main problems in the healthcare. In recent years, nosocomial infections associated with contaminated disinfectant solutions have been reported in all regions of the world. Most cases have been associated with water-based CHX, QACs, and the combination of CHX and a quaternary ammonium compound (QAC) [20]. Resistance of microorganisms to disinfectants in combination with resistance to antibiotics can lead to the spread of epidemically dangerous strains. In this regard, such microorganisms have been actively monitored recently, although not on a permanent basis [2,3]. One of the main mechanisms of bacterial defense against biocides are multidrug efflux pumps, which reduce the intracellular concentration of substances by removing them from the cell. Efflux pumps have specific substrates; however, some can pump out several different types of antimicrobial drugs, resulting in multidrug resistance [21]. Efflux pumps are involved in the modulation of bacterial behavior and virulence as well as in the maintenance of bacterial homeostasis under various stress factors in the host [22]. Therefore, it is essential to characterize the susceptibility of the ESKAPEE pathogens (*E. coli*, *K. pneumoniae*, *P. aeruginosa,* and *S. aureus*) to disinfectants and antiseptics (chlorhexidine bigluconate and a composite solution of chlorhexidine and a cationic surfactant) and antibiotics (beta-lactams, fluoroquinolones, and aminoglycosides) and to determine the prevalence of efflux pump genes associated with biocide resistance.

CHX is a broad-spectrum biocide that is effective against bacteria, fungi, and some protozoa. It is more effective against Gram-positive than Gram-negative bacteria, as the later have an outer membrane that restricts biocide penetration [17,23]. In addition, reduced biocidal efficacy may be related to a decrease in porin expression, changes in the composition of the bacterial cell membrane (proteins, fatty acids, and phospholipids), as well as changes in membrane potential [24]. Morrissey et al. reported the highest MIC values of CHX for *E. coli*, *E. faecalis*, and *K. pneumoniae* compared to other bacterial species [25]. However, Gram-positive bacteria can also be resistant to CHX. In a recent study, four *S. aureus* isolates were found to be tolerant to CHX with an MIC = 4 mg/mL (0.4%) [26]. In our study, CHX inhibited the growth of most nosocomial strains of *E. coli*, *K. pneumoniae*, *P. aeruginosa*, and *S. aureus* at low concentrations (MIC_CHX_ < 0.0008%). The same concentrations had a bactericidal effect, and the MIC was 0.0008% in most cases. The most resistant were *P. aeruginosa*, which were killed in most cases at 0.0016%. Gram-positive staphylococci were the most sensitive with an MBC_CHX_ 100 times lower than that of pseudomonads. These data are consistent with the results of other studies in which CHX inhibited the growth of all microorganisms tested (*E. coli*, *S. aureus,* and *P. aeruginosa*, including MRSA, etc.) at a concentration of 0.0004%, and lower MICs were also found for Gram-positive bacteria [27]. According to another study, CHX completely lysed cells of *E. coli* and *S. epidermidis* at a higher concentration of 0.0031% [28]. The difference in concentrations is probably not only due to the different sensitivity of the cultures, but also to differences in the media and cultivation conditions, as well as the duration of exposure to the biocide. The 0.05% CHX solutions retain their efficacy against most nosocomial strains of various bacterial species even when diluted more than 50-fold. QAC has also been shown to have better activity against Gram-positive *S. aureus* than against Gram-negative *E. coli* [29]. However, tolerance to BAC was observed in almost 30% of *S. aureus* isolates, with a MIC of 0.0032% [26]. Our study showed that S7, which contains a higher concentration of CHX and BAC, effectively suppressed the growth of most microorganisms tested even at a 4000-fold dilution.

It is known that bacteria in biofilms are less sensitive to disinfectants than in the planktonic state [30,31]. In our study, the planktonic bacteria were killed when the biocides were diluted 50- to 1000-fold, while the cells of some strains in biofilms remained alive after one hour exposure to CHX, but not to S7. The greater resistance of Gram-negative bacteria to biocides compared to Gram-positive bacteria is not only due to the presence of an outer membrane, but also to the formation of more massive biofilms [32]. In a recent study, highly biofilm-forming isolates *K. pneumoniae* and *P. aeruginosa* were obtained from CHX-based handwash during microbiological surveillance of “in-use disinfectants” in a hospital in Varanasi (India) [19]. In our study, biofilms formed by *P. aeruginosa* were the most massive (OD_570_ = 0.958 ± 0.879), while most strains were categorized as SBF and were the most tolerant to CHX compared to other species. *K. pneumoniae* biofilms were less massive (OD_570_ = 0.430 ± 0.204). *E. coli* and *S. aureus* biofilm biomasses were similar (OD_570_ = 0.154 ± 0.107 and OD_570_ = 0.154 ± 0.033, respectively), but this parameter was more variable in *E. coli. P. aeruginosa* was identified as a strong biofilm producer [33]. CHX has been shown to stimulate the expression of the *psl* operon, which encodes an exopolysaccharide important for biofilm formation in *P. aeruginosa* via the LadS/GacSA pathway (c-di-GMP independence) [31]. It should be noted that most studies on the effect of CHX and CHX-containing biocides on biofilm formation and degradation are based on the assessment of biofilm biomass after staining with 0.1% crystal violet, measured spectrophotometrically at λ = 570 nm. Coles et al. reported that CHX can lead to a significant decrease in the massiveness of the total biofilm [27]. To assess the effect of biocides on biofilms, we counted the number of viable cells in the biofilm after one hour of exposure to biocides. The complete bactericidal effect of S7 on cells in biofilms was demonstrated for bacteria of all species. In the case of CHX, viable cells were preserved, but their number decreased by 3–6 orders of magnitude, which also corresponds to a biocidal effect or “sterilizing” effect [34]. Results by Fink et al. demonstrated that CHX in concentration of 3 MIC (0.06 mg mL^−1^) decreased viability of *E. coli* (3.5 log CFU cm^−2^) and *S. aureus* (3.8 log CFU cm^−2^) in biofilm [35]. As nosocomial bacteria frequently come into contact with CHX, bacterial adaptations are possible. It has already been shown that CHX in very low concentrations can reduce the viability of planktonic cells and protect against plankton growth. Since CHX in sub-MIC doses can stimulate biofilm formation, concern over the inappropriate use of cationic disinfectants is important [31]. It should be noted that statistical analysis did not reveal statistically significant correlations between biofilm biomass and bacterial survival following exposure to biocides for any species tested.

The most common mechanism of bacterial resistance to disinfectants and antiseptics is the expression of efflux systems. The disinfectant resistance genes *qacE, qacEΔ1,* and *cepA* have been detected in Gram-negative multidrug-resistant bacteria. Qac efflux pumps belong to the SMR superfamily and are named for their ability to confer resistance to QAC-based antiseptics [16]. These pumps have been shown to be important genetic biomarkers for predicting the presence of class 1 integrons, antiseptic tolerance, and environmental QAC contamination [36]. The *qacE* and *qacEΔ1* genes were first described in *K. pneumoniae* in the 3′-conserved segment of an integron on plasmid R751 [37]. The *qacEΔ1* gene is a modified form of *qacE* resulting from the insertion of a DNA segment containing the sulfonamide resistance gene near the 3′ end of the *qacE* gene. The *cepA* gene, which encodes a cation efflux pump, is known to be associated with CHX resistance in *K. pneumoniae* [38]. In a study on the resistance of clinical *K. pneumoniae* strains to BAC, the *qacΔE1* gene was detected in 53–87.5% of bacteria [39,40]. Kosyakova et al. reported that the frequencies of *qacE*, *qacEΔ1*, and *cepA* in *K. pneumoniae* were 33.3, 23.3, and 83.3%, respectively [41]. In our study, similar results were obtained. Specifically, *qacE, qacEΔ1,* and *cepA* were prevalent in nosocomial *K. pneumoniae* with frequencies of 42.1%, 47.4%, and 87.7%, respectively. In *P. aeruginosa*, *qacE* and *qacEΔ1* were not very common (28.6% and 21.4%, respectively). Low frequencies of the genes *qacE* (2.7%) and *qacEΔ1* (10%) in *P. aeruginosa* were previously documented in Germany [42]. In multidrug-resistant *P. aeruginosa*, the *qacE* and *qacEΔ1* genes were amplified in 1 (1.1%) and 34 (36.9%) isolates, respectively, in a recent study [43]. Other studies report higher prevalence of these genes. For example, *qacEΔ1* was detected in 213 of 331 (64.4%) clinical isolates of *P. aeruginosa* in China [44]. In the work of Radmeh et al., the *qacE* and *qacEΔ1* genes were detected simultaneously in 66% of isolates [45]. Previously, Kazama et al. identified the *qacEΔ1* and *qacE* genes in 41 (65.1%) and 15 (23.8%) strains of clinical *P. aeruginosa*, respectively, while 14 of 15 strains with the *qacE* gene also possessed the *qacEΔ1* gene [46]. In our study, 62.5% of *qacE*+ cultures also had *qacEΔ1*. In silico PCR showed that the primer for *qacE* does not anneal in the *qacEΔ1* region, so we assume that these genes are indeed located in the 3′ segment of various integrons in the strains studied, as previously suggested by Kazama et al. [46]. Genes of the *qac* group have been shown to be frequently detected in association with genes for resistance to antibiotics of various groups, including β-lactams [42]. In our study, the *qacE* gene was only found among MDR *K. pneumoniae* and significantly more often among MDR *P. aeruginosa*. In MDR *E. coli* and *K. pneumoniae*, *qacEΔ1* was also significantly more frequent.

The three-component pumps OqxAB, AcrAB-TolC, and MexAB-OprM belong to the RND superfamily, the most important family of efflux pumps in Gram-negative bacteria. Recently, the role of the OqxA and OqxB pumps has increased. The *oqxA* and *oqxB* genes can be located on plasmids and are often associated with resistance to fluoroquinolones, tigecycline, QACs, and biguanide disinfectants [38,46]. Previously, Yuan et al. found *oqxAB* genes in all *K. pneumoniae* strains studied, but only in 6.6% of *E. coli* [47]. A lower frequency of these determinants in *K. pneumoniae* was reported by Dehnamaki et al. Specifically, 57% and 56% of isolates (n = 100) had the *oqxA* and *oqxB* genes, respectively [48]. In the study by Ni et al., the frequency of *oqxA* and *oqxB* in *K. pneumoniae* was 60.9% and 17.2%, respectively [49]. In our study, these genes were also detected in many of *K. pneumoniae* strains, with 77.2% harboring *oqxA* and 82.5% harboring *oqxB*. However, these genes were rarely found in *E. coli* (11.5% and 0%, respectively), while *oqxA* was only found in strains with MBC CHX ≥ 2%. The differences in the reported prevalence could be due to methodological differences (e. g. different sample sizes, isolates from different sources, clonality of isolates), but also to geographical factors. The AcrAB-TolC multidrug efflux pump system controls the release of antibiotics, oil solvents, and biocides, including CHX [50]. Several studies have found that the presence of the AcrAB-TolC pump, along with other resistance mechanisms, contributed to a reduction in the MIC of antibiotics and the emergence of multidrug resistance [51,52]. We detected AcrAB-TolC pump determinants in 87.7% of *K. pneumoniae* and 100% of *E. coli* strains. This pump is indeed a widespread resistance mechanism in *E. coli* [53]. Mutations in genes encoding RND efflux pumps such as *marA* lead to upregulation of the AcrAB-TolC RND protein pumps by overexpression of the MarA protein in *E. coli* [54]. The presence of *acrA*, which encodes a protein that links two integral membrane proteins of the AcrAB-TolC pump, provides tolerance of *K. pneumoniae* to various antibiotics, including fluoroquinolones, as well as to biocides, including ethanol, chlorhexidine, and benzalkonium bromide [55]. The AcrAB efflux pump not only contributes to the multidrug resistance phenotype, but may also be a novel virulence factor required by *K. pneumoniae* to resist immune defense mechanisms in the lung, contributing to the development of pneumonia [56]. The pleiotropic role of AcrAB-TolC in *K. pneumoniae* pathobiology has been demonstrated. In addition to targeting antimicrobial resistance, TolC plays a regulatory role in capsule biosynthesis, iron homeostasis, adherence to host cells, sensitivity to serum, and virulence (the *tolC* mutant displayed reduced virulence compared to the wild type in the *Galleria mellonella* infection model) [57]. In our study, all *K. pneumoniae* strains contained *acrA*, while other studies reported that only 19% of carbapenem-resistant *K. pneumoniae* possessed this gene [55]. In our study *oqxA* was detected only in one *P. aeruginosa* strain, while *oqxB*, *acrA*, and *acrB* were not detected. In contrast, the MexAB-OprM pump was detected in 50% of *P. aeruginosa* strains and was not found in other Gram-negative bacteria. The obtained data are consistent with literature sources. Aparna et al. describe the antibiotic resistance of *E. coli* and *P. aeruginosa* due to the work of the efflux pumps AcrAB-TolC and MexAB-OprM, respectively [58]. A structural correlation has been demonstrated between MexAB *P. aeruginosa* and AcrAB *E. coli*, which are integral membrane proteins that are part of three-component efflux systems [58]. The high degree of homology between MexAB and AcrAB (57.7% identity MexA-AcrA; 69.8% identity MexB-AcrB) enabled cloning and provision of MexAB-OprM function in *E. coli* without a significant decrease in the activity spectrum. Nevertheless, the resistance level of *E. coli* was significantly lower than that of *P. aeruginosa*, even to the agents to which resistance is provided by MexAB-OprM, probably reflecting the higher permeability of the outer membrane of *E. coli* compared to *P. aeruginosa* [59].

All 29 *S. aureus* isolates were analyzed for the presence of six chromosomally encoded genes (*norA*, *norB*, *norC*, *sepA*, *mepA*, and *mdeA*) and two plasmid encoded genes (*qacA/B* and *smr*) encoding efflux pumps in *S. aureus*. The removal of disinfectants and antiseptics from the cell is mediated by the *qacA/B* and *smr* genes in Gram-positive bacteria, particularly in staphylococci [60]. The *qacA/B* gene is responsible for resistance to various organic cations, including BAC, ethidium bromide, cetrimide, and CHX. The spread of *qacA/B* among *S. aureus* and other members of the genus *Staphylococcus* is facilitated by horizontal gene transfer between strains via the conjugative plasmid [61]. Small genes of multidrug resistance, including *smr* (staphylococcal multidrug resistance, known as *qacC/D*), are also responsible for resistance to antiseptics in staphylococci [62]. According to various authors, the *qacA/B* and *smr* genes were detected in 0–95% of clinical *S. aureus* strains, indicating heterogeneity in the presence of these genes in the population. Damavandi et al. reported 12.5% of *S. aureus* isolates carried the *qacA/B* gene and 31.7% of isolates carried the *smr* gene [62]. According to Schlett et al., only 1.6% of 615 isolates were *qacA/B*-positive [63]. In the study of McClure et al., none of the 296 screened *S. aureus* isolates contained both the *qac* and *smr* genes [64]. According to Ghasemzadeh-Moghaddam et al., the *qacA/B* gene was present in 68% of methicillin-resistant *S. aureus* (MRSA) and 58.2% of methicillin-sensitive *S. aureus* (MSSA), and the *smr* gene was present in 39% and 29.3% of strains, respectively [65]. The efflux pump genes described above are frequently identified in *Staphylococcus* isolates that exhibit reduced susceptibility to CHX; however, none of these genes have been detected in MRSA strains with BAC resistance [66]. The absence of *qacA/B* and *smr* genes in CHX-susceptible strains is quite understandable; however, these genes were found by us in some strains of other *Staphylococcus* species.

The proteins NorA, NorB, NorC, and MdeA belong to the MFS family; MepA belongs to the MATE family; and SepA belongs to the SMR family [67]. Reduced susceptibility to antibiotics, biocides, and dyes can be indirectly associated with increased expression of these pumps in staphylococci. NorA enables efflux of a wide range of biocides, including QACs and antibiotics, with some fluoroquinolones being among the best substrates for transport [14,68]. Expression of Nor efflux pumps in *S. aureus* is controlled by the global regulator MgrA (multiple gene regulator). MgrA can be phosphorylated post-translationally by the putative serine/threonine kinase PknB, and the phosphorylated MgrA-P can be dephosphorylated by RsbU, which alters the ability of MgrA to bind to the *norA/B* promoters. Phosphorylation of MgrA results in loss of binding to the *norA* promoter and binding to the *norB* promoter. Loss of the ability to phosphorylate MgrA resulted in increased repression of *norA* expression and decreased susceptibility to NorA substrates, including antibiotics and disinfectants [69,70]. It should also be noted that NorA can contribute to the export of siderophores [71]. Exposure of *S. aureus* isolates to low concentrations of biocides resulted in increased expression of the *norA*, *norC*, *mepA*, and *mdeA* genes and increased resistance to biocides and antibiotics, including CHX [17]. Overexpression of *norB* leads to resistance to fluoroquinolones, tetracycline, and even disinfectants and dyes, while overexpression of *mepA* leads to resistance to fluoroquinolones and disinfectants [68]. The prevalence of *norA*/*norB*/*norC* genes in *S. aureus* isolates often exceeds 50% [72]. The multidrug resistance efflux pump SepA (antiseptic resistance protein SepA/staphylococcal efflux pump A) is involved in multicomponent efflux in staphylococci. It provides low resistance to drugs such as acriflavine, BAC, chlorhexidine gluconate, and ethidium bromide [73]. The protein SepA, encoded by the chromosomal gene *sepA*, is a transporter consisting of 157 amino acids and four putative transmembrane segments, which is typical for transporters of the SMR family. However, SepA lacks conserved motifs of this family. Although some residues important for H+:drug transport specificity and antiport are present elsewhere, SepA may belong to an as of yet unidentified transporter family. Antiabong et al. assessed the diversity of all above genes in clinical *S. aureus* isolates obtained at Pretoria hospital (South Africa) and the prevalence of all multidrug efflux pump genes in *S. aureus* was very high at 79–99% [74]. In strains analyzed in our study, efflux pump genes were prevalent at the level of other studies.

Microbial adhesion on abiotic surfaces and thus biofilm formation are considered a serious problem, both in terms of economic and public health consequences. In recent decades, the healthcare environment (medical devices and surfaces, commonly referred to as “fomites”) has been increasingly recognized as a reservoir of bacteria that cause healthcare-associated infections [75,76]. These bacteria can be transmitted from fomites to patients through direct contact, through contact with (semi-)critical object, and via the hands [8]. Indeed, representatives of all the taxa studied were preserved for 24 h on the surface of “ceramic tiles” and “plastic”. Nevertheless, under conditions close to the in situ situation, the complete bactericidal effect of CHX and S7 on the cells of all tested strains was demonstrated on the surface in the immediate (except for *P. aeruginosa*) and daily exposure variants of the experiment.

Today, mechanisms that reduce the sensitivity of microorganisms to disinfectants and antibiotics are known, e.g., the release of biocides from the cell [50,77], the mucoid phenotype, and biofilm formation [78]. We found no correlation between biofilm biomass and bacterial survival after exposure of daily biofilms to biocides. The presence of individual efflux pump genes differed significantly in the groups of strains with different MBC_CHX_: *oqxA* was found only in *E. coli* strains with MBC_CHX_ ≥ 1%; *qacE*, *qacEΔ1*, and a larger number of efflux pump genes were more often contained in *P. aeruginosa* strains with MBC_CHX_ ≥ 2%. We found no significant correlations between antibiotic resistance and tolerance to chlorhexidine-based biocidal compositions. However, in our study, MDR strains contained more efflux pump genes per strain (*K. pneumoniae*) or more often contained three efflux pump genes simultaneously than non-MDR strains of *E. coli* and *P. aeruginosa*.

It is known that subinhibitory concentrations of disinfectants can also contribute to the horizontal transfer of antibiotic resistance genes [79]. There is evidence that the use of QACs and sulfonamides since the 1930s has contributed to the spread of class 1 integrons and, thus, to the development of antibiotic resistance in clinically significant bacteria [80]. After testing *K. pneumoniae* strains for their resistance to antibiotics and biocides and finding the presence of integrons in them, Samir et al. concluded that the presence of class 1 integrons in MDR *K. pneumoniae* isolates may only contribute to some biocide resistance, but does not appear to be the only factor important for multiple drug resistance [81]. However, some authors find no evidence of combined resistance to disinfectants and antibiotics [82]. In this regard, additional data and studies are needed to confirm that the use of disinfectants in hospitals contributes to the emergence of bacterial resistance to biocides.

## 4. Materials and Methods

### 4.1. Bacterial Strains

In this study, non-clonal bacterial strains of *E. coli* (n = 26), *K. pneumoniae* (n = 57), *P. aeruginosa* (n = 23), and *S. aureus* (n = 29) isolated from sputum, bronchopulmonary lavage, blood, wound, and urine of hospitalized patients of surgical departments of multidisciplinary hospitals in Perm, Russia (n = 6), during the period of 2018–2023 were used. For microbial identification, traditional culture-based methods and mass spectrometry, including MALDI TOF SM, were used in the laboratories of local hospitals. All strains were stored in the departmental collection of Perm State Medical University Named After Academician E. A. Wagner. The reference strains of *E. coli* ATCC^®^25922, *K. pneumoniae* ATCC^®^700603, *P. aeruginosa* ATCC^®^27853, and *S. aureus* ATCC^®^25923 used in this study were obtained from the collection of the State Research Institute for Standardization and Control of Medical Biological Preparations, named after L.A. Tarasevich (Moscow) [83].

### 4.2. Antimicrobial Susceptibility Testing

The antimicrobial susceptibility of the bacterial strains was determined according the clinical guidelines outlined in the “Determination of the sensitivity of microorganisms to antimicrobial drugs” of the Interregional Association for Clinical Microbiology and Antimicrobial Chemotherapy (IACMAC, Version-2018-03). The strains were tested on Muller–Hinton agar (“FBIS SRCAMB”, Obolensk, Russia) using the disc diffusion method with discs (“NICF”, St. Petersburg, Russia). Bacterial strains that were resistant to at least one antibiotic from three or more antibiotic groups were defined as multidrug resistant (MDR) strains [84].

### 4.3. Biocidal Compositions

In this study, a 0.05% chlorhexidine bigluconate (CHX) solution (Samaramedprom OJSC, Russia) and the biocidal composite solution S7 (S7) with 0.07% chlorhexidine and 0.055% cationic surfactant benzalkonium chloride (BAC), also known as alkyldimethylbenzylammonium chloride (ADBAC) (Institute of Technical Chemistry, Perm, Russia), were used (certificate of state registration № RU 77.99.88.002.E.004217.12.20, date 10.12.2020: Eurasian Economic Union Federal Service for Supervision of Consumer Rights Protection and Human Wellbeing).

### 4.4. Assessment of the Effect of Biocides on Planktonic Cells (MIC, MBC)

The MICs of the biocidal compositions were determined using the broth dilution method. Cells were grown overnight at 37 °C in a glass tube containing 5 mL Muller–Hinton broth. The next day, overnight cultures were diluted 1:100 in fresh medium and cultured for 5 h. The cultures were then adjusted to an OD of 0.1 (A625), diluted 1:100, and added to serial dilutions of the biocidal compositions in 96-well plates. The plates were incubated for 24 h at 37 °C without shaking. After the 24-h incubation, the MIC was determined. To determine the MBC, 10 µL from each well used for the MIC determination was inoculated on a Petri dish with Muller–Hinton agar and incubated for 24 h at 37 °C. The MBC was determined as the lowest concentration of an antimicrobial agent required to prevent colony growth. The concentration of the initial biocidal solution (CHX or S7) was taken as 100%, and a series of two-fold serial dilutions was prepared. MIC and MBC were calculated as % of the initial solution.

### 4.5. Biofilm Biomass

Overnight cultures of the strains were diluted 100-fold in LB broth (Amresco, Solon, OH, USA), and 200 μL was added to the wells of a 96-well flat bottom polystyrene plate (Medpolimer, Saint Petersburg, Russia) and incubated for 24 h at 37 °C without shaking. The biofilm biomass was determined according to Merritt et al. [85]. The biofilms formed were washed twice with NaCl (0.9%), stained with 0.1% crystal violet for 30 min, and washed three times with distilled water. The dye was extracted with 200 μL of 96% ethanol to determine the biofilm biomass by optical density (OD_570_) using an Infinite M1000 (TECAN, Grödig, Austria). The experiments were repeated three times with six technological replicates. Based on the OD of the negative control, the cutoff value (ODc) was calculated as the OD of the negative control plus 3 × standard deviation (SD) of the negative control. Based on ODc, the strains were divided into four groups, including strains without biofilm formation (NBF) (ODo < ODc), strains with weak biofilm formation (WBF) (ODc < OD < 2ODc), strains with moderate biofilm formation (MBF) (2ODc < OD < 4ODc), and strains with strong biofilm formation (SBF) (4Odc < OD) [86].

### 4.6. Evaluation of the Effect of Biocidal Compositions on the Cells in a Biofilm

Overnight cultures of nosocomial strains were adjusted with fresh LB broth to OD of 0.1 at 600 nm (UV-1680 spectrophotometer, Shimadzu, Kyoto, Japan). This cell suspension was diluted 10-fold with LB broth, and 100 μL was added to the wells of 96-well plates. The plates were incubated for 24 h at 37 °C without shaking. Then, the liquid culture was removed, and 150 μL of CHX/S7/NaCl was added to the wells and incubated for 1 h at 37 °C. The solutions were then removed. The wells were washed three times with sterile saline, and 100 μL of saline was added. Then, the plates were then sonicated (5 times for 1 min at 37 kHz) in an Elma Ultrasonic 30S ultrasonic bath (Elma, Berlin, Germany). Ten-fold dilutions of the sonicated culture were then plated on an agar nutrient medium and incubated for 24 h at 37 °C. After incubation, the CFU was determined, and the effectiveness of the disinfectant against biofilm was assessed according to [34]. The term “disinfectant” refers to a biocidal effect that results in a reduction in the titer/number of CFU by at least three orders of magnitude (99.9%). The term “sterilizing” refers to either the complete destruction of microorganisms or a reduction in the titer/CFU by six orders of magnitude or more.

### 4.7. Evaluation of Antimicrobial Activity of Biocidal Compositions on Surfaces

The antimicrobial activity of biocidal compositions on surfaces was evaluated in accordance with GOST R 58151.4-2018 and methodological recommendations of Nizhny Novgorod, 2010 [87,88]. Bacterial cultures were grown for 18–24 h at 37 °C in LB broth and then diluted 100-fold in 0.9% NaCl. The surfaces to be tested were pre-cleaned and sterilized with 70% ethanol. Then, 0.1 mL of bacterial suspension was applied to the surface (10 × 10 cm^2^) and spread evenly with a sterile spatula for 1 min. Then, 0.9 mL of CHX/S7/NaCl was applied, spread evenly with a sterile spatula, and incubated (10, 30, and 60 min). Then, 0.5 mL of neutralizer was added, and the mixture was immediately seeded on an agar nutrient medium with a sterile cotton swab. The surface contamination, the completeness of neutralizer action, and the purity of disinfectant solutions and the neutralizer were monitored. The plates were incubated at 37 °C for 24 h. In the second series of experiments, the bacterial suspension was applied to the selected surface and left for 24 h. Then, the biocidal compositions were applied as described above. The sensitivity of the strains and the efficacy of the biocide were evaluated in a similar manner. The evaluation of the antimicrobial activity of the biocidal compositions was performed on the surfaces of “ceramic tiles” and “plastic” (polystyrene) without additional treatment. From nosocomial strains of *E. coli*, *K. pneumoniae*, *P. aeruginosa,* and *S. aureus,* one random strain with average values of the main parameters (*E. coli* EC8, *K. pneumoniae* KP20, *P. aeruginosa* PA5, and *S. aureus* SA12) was selected to evaluate the effect of biocidal compositions on surfaces.

The susceptibility of the strain was evaluated by determining the CFU number. The strain was considered resistant to the disinfectant with a growth of 300 CFU/mL or more. The strain was considered susceptible when no growth or a growth of no more than 300 CFU/mL was observed, which corresponds to the required efficacy of the disinfectant (killing of 99.99% of microorganisms). Strains with less than 300 CFU/mL were grouped according the degree of susceptibility: (1) complete sensitivity—with no growth; and (2) incomplete sensitivity: (i) 1–99 CFU/mL—incomplete bactericidal action, and (ii) 100–299 CFU/mL—sub-bactericidal action.

### 4.8. PCR Detection of Efflux Pump Genes

To obtain matrix DNA for PCR amplification, a loop of bacterial biomass was resuspended in 100 µL ultrapure water, heated for 15 min at 97 °C in a solid-state thermostat with a timer TT-2 “Termit” (Moscow, Russia), and centrifuged for 5 min at 13,000 rpm in a microcentrifuge 5415R (“Eppendorf”, Hamburg, Germany). The supernatants were transferred to fresh Eppendorf tubes and stored at –20 °C until further use. Gram-negative bacteria were analyzed by PCR for the presence of the following efflux pump genes: *qacEΔ1, qacE, acrAB, oqxA*, *oqxB*, and *mexA/B*. In addition, *K. pneumoniae* strains were analyzed for the presence of the *cepA* gene encoding the chlorhexidine efflux pump. All staphylococci were analyzed for the presence of efflux pump genes: *qacA/B*, *norA*, *mepA*, *mdeA*, *norB*, *norC*, and *sepA*.

All primers used were synthesized at the LLC “Sintol” (Moscow, Russia). The nucleotide sequences of the primers, the PCR programs, and the amplicon sizes are given in Table 4. Amplifications were carried out in PCR mixtures containing 3 μL DNA template, 0.4 μL 5 U/mL Taq-polymerase, 2.5 μL 10× PCR buffer, 2.5 μL 25 μM MgCl_2_, 0.25 μL 25 μM dNTPs, and 2.5 μL 10 µM forward and reverse primers (LLC “Sintol”, Moscow, Russia) in a total volume of 25 μL in a thermal cycler DNA Engine Dyad Thermal Cycler (“Bio-Rad”, Foster City, CA, USA). After amplification, 10 µL of each PCR reaction was separated on a 1.2% agarose gel electrophoresis using Agarose E (“diaGene”, Novosibirsk, Russia), and stained with ethidium bromide (0.5 mg/mL). Band visualization and data documentation were performed with the gel documentation system Gel-DocXR (“Bio-Rad”, Foster City, CA, USA).

### 4.9. Statistical Analysis

Data were expressed as mean and standard deviation (mean ± SEM) or median (Me). Statistical analysis was performed using Student’s *t*-test or Fisher’s exact test to compare qualitative characteristics. Spearman’s rank correlation test was used to evaluate the correlation between phenotypic and genotypic characteristics. A *p*-value of less than 0.05 was considered significant. Statistical analysis was performed using Excel or GraphPad Prism 8 Version 8.0.1 (GraphPad Software, Boston, MA, USA). The GraphPad Prism 8 software package was used for plotting graphs.

## 5. Conclusions

The activity of CHX and the biocidal composite solution based on CHX and QAC (S7) on nosocomial strains of *E. coli*, *K. pneumoniae*, *P. aeruginosa*, and *S. aureus* was investigated in several models. It was shown that CHX and S7 in low concentrations have a bacteriostatic and bactericidal effect on planktonic cells of most nosocomial strains. *S. aureus* strains were the least resistant to biocides, while *P. aeruginosa* showed the highest resistance. A study of the effect on biofilms showed high activity of both biocides, with S7 being more effective than the standard 0.05% CHX. Determinants of four families (RND, SMR, MFS, and MATE) of efflux pumps were identified. MDR Gram-negative bacteria (*E. coli*, *K. pneumoniae* and *P. aeruginosa*) contained more efflux pump genes. Although the biocide concentrations used are still well above the MIC or MBC for biocides in bacteria with reduced susceptibility to biocides, this should not lead to a decrease in attention to this problem.

## Figures and Tables

**Table 1:** Comparison of the survival of bacteria *E. coli*, *K. pneumoniae*, *P. aeruginosa*, and *S. aureus* under the action of CHX and S7 in plankton and biofilm.

Parameter	Microorganism			
	*E. coli* (n = 26)	*K. pneumoniae* (n = 57)	*P. aeruginosa* (n = 23)	*S. aureus* (n = 29)
Median MIC and MBC in plankton culture, final concentration of CHX or S7 (CHX/BAC) in %				
MIC_CHX_	0.00038	0.00078	0.00078	0.000006
MBC_CHX_	0.00078	0.00078	0.00156	0.000025
MIC_S7_	0.00000013/ 0.00000011	0.0000084/ 0.0000066	0.000017/ 0.000013	0.00000013/ 0.0000001
MBC_S7_	0.00000027/ 0.00000021	0.0000084/ 0.0000066	0.000017/ 0.000013	0.00000026/ 0.00000021
Bacterial survival in % (median CFU/mL) in one-day old biofilm after application of the biocidal solution				
After CHX	80.8 (7.98 × 10^3^)	5.3 (3.3 × 10^2^)	32.1 (1.0 × 10^1^)	34.5 (0.7 × 10^1^)
After S7	0	0	0	0
Biofilm biomass, median OD_570_	0.123	0.391	0.703	0.158

Note. MIC—minimum inhibitory concentration, MBC—minimum bactericidal concentration, CHX—chlorhexidine, S7—composite solution of CHX (0.07%) and the cationic surfactant benzalkonium chloride (BAC, 0.055%).

**Table 2:** Comparison of the survival of randomly chosen strains of *E. coli*, *K. pneumoniae*, *P. aeruginosa*, and *S. aureus* with the immediate action of CHX and S7 on the “ceramic tile” and “plastic” surfaces.

Exposure Time, min	Solution	*E. coli* Strain EC8		*K. pneumoniae* Strain KP20		*P. aeruginosa* Strain PA5		*S. aureus* Strain SA12	
		CFU/100 cm^2^	Effect	CFU/100 cm^2^	Effect	CFU/100 cm^2^	Effect	CFU/100 cm^2^	Effect
Ceramic tiles									
10	NaCl	>300	NBE	>300	NBE	>300	NBE	>300	NBE
	CHX	0	Bactericidal	0	Bactericidal	50–99	IBA	<5	IBA
	S7	0	Bactericidal	0	Bactericidal	0	Bactericidal	0	Bactericidal
30	NaCl	>300	NBE	>300	NBE	>300	NBE	>300	NBE
	CHX	0	Bactericidal	0	Bactericidal	0	Bactericidal	0	Bactericidal
	S7	0	Bactericidal	0	Bactericidal	0	Bactericidal	0	Bactericidal
60	NaCl	>300	NBE	>300	NBE	>300	NBE	>300	NBE
	CHX	0	Bactericidal	0	Bactericidal	0	Bactericidal	0	Bactericidal
	S7	0	Bactericidal	0	Bactericidal	0	Bactericidal	0	Bactericidal
Plastic									
10	NaCl	>300	NBE	>300	NBE	>300	NBE	>300	NBE
	CHX	0	Bactericidal	0	Bactericidal	5-99	IBA	0	Bactericidal
	S7	0	Bactericidal	0	Bactericidal	0	Bactericidal	0	Bactericidal
30	NaCl	>300	NBE	>300	NBE	>300	NBE	>300	NBE
	CHX	0	Bactericidal	0	Bactericidal	0	Bactericidal	0	Bactericidal
	S7	0	Bactericidal	0	Bactericidal	0	Bactericidal	0	Bactericidal
60	NaCl	>300	NBE	>300	NBE	>300	NBE	>300	NBE
	CHX	0	Bactericidal	0	Bactericidal	0	Bactericidal	0	Bactericidal
	S7	0	Bactericidal	0	Bactericidal	0	Bactericidal	0	Bactericidal

Note. NBE—no bactericidal effect, IBA—incomplete bactericidal action.

**Table 3:** Comparison of the survival of randomly chosen strains of *E. coli*, *K. pneumoniae*, *P. aeruginosa*, and *S. aureus* under the action of CHX and S7 on one-day old biofilms formed on the surface of “ceramic tile” and “plastic”.

Exposure Time, h	Solution	*E. coli* Strain EC8		*K. pneumoniae* Strain KP20		*P. aeruginosa* Strain PA5		*S. aureus* Strain SA12	
		CFU/ 100 cm^2^	Effect	CFU/ 100 cm^2^	Effect	CFU/ 100 cm^2^	Effect	CFU/ 100 cm^2^	Effect
Ceramic tiles									
1	NaCl	>100	NBE	>300	NBE	10–50	NBE	>100	NBE
	CHX	0	Bactericidal	0	Bactericidal	0	Bactericidal	0	Bactericidal
	S7	0	Bactericidal	0	Bactericidal	0	Bactericidal	0	Bactericidal
2	NaCl	>100	NBE	>300	NBE	10–50	NBE	>100	NBE
	CHX	0	Bactericidal	0	Bactericidal	0	Bactericidal	0	Bactericidal
	S7	0	Bactericidal	0	Bactericidal	0	Bactericidal	0	Bactericidal
Plastic									
1	NaCl	10–50	NBE	>300	NBE	10–50	NBE	>100	NBE
	CHX	0	Bactericidal	0	Bactericidal	0	Bactericidal	0	Bactericidal
	S7	0	Bactericidal	0	Bactericidal	0	Bactericidal	0	Bactericidal
2	NaCl	10–50	NBE	>300	NBE	10–50	NBE	>100	NBE
	CHX	0	Bactericidal	0	Bactericidal	0	Bactericidal	0	Bactericidal
	S7	0	Bactericidal	0	Bactericidal	0	Bactericidal	0	Bactericidal

Note. NBE—no bactericidal effect.

**Table 4:** The primer sequence, PCR program, and expected amplicon size used in the study.

Gene	Nucleotide Sequence (5′–3′)	PCR Program	Amplicon Size (bp)	Reference
*qacEΔ1*	TAGCGAGGGCTTTACTAAGC ATTCAGAATGCCGAACACCG	93 °C, 2 m; 35 [93 °C, 30 s; 55 °C, 30 s; 72 °C,1 m]; 72 °C, 10 m	300	[89]
*qacE*	CCCGAATTCATGAAAGGCTGGCTT AAGCTTTCACCATGGCGTCGG			
*cepA*	CAACTCCTTCGCCTATCCCG TCAGGTCAGACCAAACGGCG	94 °C, 5 m; 30 [94 °C, 30 s; 53 °C, 1 m; 72 °C, 2 m]; 72 °C, 7 m	1051	
*oqxA*	CTCGGCGCGATGATGCT CCACTCTTCACGGGAGACGA	95 °C, 1 m; 35 [95 °C, 45 s; 60 °C, 45 s; 72 °C, 1 m]; 72 °C, 5 m	392	[48]
*oqxB*	TTCTCCCCCGGCGGGAAGTAC CTCGGCCATTTTGGCGCGTA		512	
*acrAB*	ATCAGCGGCCGGATTGGTAAA CGGGTTCGGGAAAATAGCGCG	94 °C, 5 m; 30 [94 °C, 1 m; 56 °C, 30 s; 72 °C, 1 m]; 72 °C, 10 m	312	[90]
*mexA/B*	TGTCGAAGTTTTTCATTGATAG AAGGTCACGGTGATGGT	94 °C, 3 m; 32 [94 °C, 30 s; 57 °C, 45 s; 72 °C, 1 m]; 72 °C, 5 m	280	[91]
*smr (qacC/D)*	GCCATAAGTACTGAAGTTATTGGA GACTACGGTTGTTAAGACTAAACCT	95 °C, 5 m; 35 [94 °C, 40 s; 54 °C, 50 s; 72 °C, 50 s]; 72 °C, 5 m	195	[92]
*qacA/B*	CTATGGCAATAGGAGATATGGTGT CCACTACAGATTCTTCAGCTACATG	94 °C, 5 m; 30 [94 °C, 30 s; 53 °C, 30 s; 72 °C, 1 m]; 72 °C, 5 m	416	[89]
*norA*	ATGAATAAACAGATTTTTGT CTACATATTTTGTTCTTTCA	94 °C, 3 m; 35 [94 °C, 1 m; 50 °C, 45 s; 72 °C, 1,5 m]; 72 °C, 3,5 m	1167	[71]
*norB*	TCGCCTTCAACACCATCAAC GGCGTAGGAGATGATGGTCA	94 °C, 3 m; 35 [94 °C, 1 m; 52 °C, 1 m; 72 °C, 1 m]; 72 °C, 3,5 m	236	[72]
*norC*	GCGGGAGTGTGTTCTTCATC CTGGAGGAAGGTGTTGAAGC	94 °C, 3 m; 35 [94 °C, 1 m; 62 °C, 45 s; 72 °C, 1,5 m]; 72 °C, 3,5 m	441	
*mepA*	GCAGTTATCATGTCTATCGGCG TGCACCTTGTAAAATGGCCA		240	[93]
*mdeA*	TATGGCGATTGTTGTTTTTACTAC AACCGTGTGCATTCATTTCTGG	94 °C, 3 m; 35 [94 °C, 1 m; 62 °C, 45 s; 72 °C, 1 m]; 72 °C, 3,5 m	1072	[94]
*sepA*	GCAGTCGAGCATTTAATGGA ACGTTGTTGCAACTGTGTAAGA	94 °C, 4 m; 35 [94 °C, 30 s; 57 °C, 55 s; 72 °C, 1 m]; 72 °C, 5 m	103	[95]

## Data Availability

Data are contained within the article or Supplementary Materials.

## Supplementary Materials

The following supporting information can be downloaded at: https://www.mdpi.com/article/10.3390/ijms26010355/s1.
